# Demande en Interdiction

**Published:** 1848-07-01

**Authors:** 


### DEMANDE EN INTERDICTION.

The *Demande en Interdiction* is a proceeding somewhat similar to a commission of lunacy. The subject in this case, which was tried on the 23rd of May, before the Tribunal Civil de la Seine, was Madame L——, and the proceedings were instituted by her son-in-law, M. B——. The facts relied upon principally in support of the process were, that the disease was hereditary, her grandfather having died mad, her brother having destroyed himself, one cousin having died in a private asylum, and another being even then in the Bicêtre. She herself had been several times confined in lunatic asyla during the early portion of her life. Her marriage, which took place when she was very young, was an unfortunate one in every respect. Mental alienation, it was alleged, showed itself very clearly in 1829, after she had undergone a painful operation, and has continued ever since, with but few and short intervals of reason. She lived in a world of chimeras and falsehoods; she became one of the most devoted followers of the celebrated Le Normand and Moreau, gave up her days and nights to the study of the pretended secrets of the magical art, and dissipated her property among these quacks, who profited largely by her credulity. Anxious to get rid of her husband, she attempted to destroy him by poison, failing which she twice endeavoured to set her apartment on fire; being defeated in that also, she then sought to commit suicide, but was again prevented. Overcome by shame and remorse, she then sought a refuge in the Couvent des Dames Repenties, where a religious exaltation soon replaced the previously existing delirium. On her withdrawal from the convent, she was confined for four years in different lunatic asyla, which she left only for a short time in 1836, having then manifested some signs of improvement. In 1837, she was taken to Charenton, the symptoms of insanity being more marked and more violent than before; thence she was removed to Dr. Pinel's; from his house to that of Dr. Baron; thence to the Salpétrière; and thence again to Dr. Baron's. The death-bed of her father, to which she was summoned, failed to rouse the glimmering spark of reason; her extravagances on that occasion were of the most extraordinary kind. Some kind of change, however, took place soon afterwards, and Madame L—— again, for a time, mingled with society, committing, however, occasionally those extravagances which indicate *une esprit dereglee.* In 1842, she became afflicted with amorous monomania; she attacked first her notary, who was about to be married, but, failing in her endeavours, she next made love to a lawyer's clerk, her correspondence with whom is reproduced in the procés. The excitement attendant on these proceedings was soon followed by delirium, and she again became an inhabitant of M. Baron's lunatic asylum. While there, her conduct was most extraordinary; she attempted to strangle the servants; flung bottles at their heads; went by night to throw snuff in their eyes; twice she endeavoured to set her bed on fire, and passed her time in screaming, crying, and singing. From this she again for a time recovered, but in 1847 she suffered a relapse, and in consequence the present *Demande en Interdiction* was made.

The counsel for the alleged lunatic asserted that the eccentricities of which she had been guilty had been caused by the conduct of her husband, and by her domestic griefs, and that she was then in full possession of her intellects.

The court decided that she should be examined by another commission, and deferred the further prosecution of the case for six months.—*Gazette des Tribunaux.*

